# Research Status of SARS-CoV-2 on Cardiovascular System Injury in Children

**DOI:** 10.21470/1678-9741-2020-0192

**Published:** 2021

**Authors:** Yuhai Zhang, Liang Wang, Shixiong Wei

**Affiliations:** 1 Department of Cardiothoracic Surgery, Baotou Clinical Medical College affiliated to Inner Mongolia Medical University, Baotou, Inner Mongolia, People’s Republic of China.; 2 Department of Cardiovascular Surgery, Chinese PLA General Hospital, Beijing, People’s Republic of China.

**Keywords:** SARS-CoV-2, Children, Cardiovascular System

## Abstract

In December 2019, the severe acute respiratory syndrome coronavirus 2 (SARS-CoV-2) began to break out in the Hubei Province of China. At present, the epidemic situation in the world continues and the number of confirmed cases is increasing every day. A recent review showed that children under the age of ten years make up about 1% of the infected population, which cannot be ignored. Studies have shown that after SARS-CoV-2 infection children can show clinical symptoms of cardiovascular system damage in addition to typical respiratory symptoms. This article mainly discusses the possible damage of SARS-CoV-2 to children's cardiovascular system and related mechanisms.

**Table:** 

Abbreviations, acronyms & symbols	
**ACE**	**= Angiotensin converting enzyme**
**ACE-1**	**= Angiotensin converting enzyme-1**
**ACE-2**	**= Angiotensin converting enzyme-2**
**ACEI**	**= Angiotensin converting enzyme inhibitor**
**Ang**	**= Angiotensin**
**ARB**	**= Angiotensin II receptor blocker**
**ARDS**	**= Acute respiratory distress syndrome**
**AT1R**	**= Angiotensin II type I receptor**
**CK**	**= Creatine kinase**
**CK-MB**	**= Creatine kinase isoenzyme**
**CT**	**= Computed tomography**
**ICU**	**= Intensive care unit**
**IL**	**= Interleukin**
**MERS-CoV**	**= Middle East respiratory syndrome coronavirus**
**RNA**	**= Ribonucleic acid**
**SARS-CoV**	**= Severe acute respiratory syndrome coronavirus**
**SARS-CoV-2**	**= Severe acute respiratory syndrome coronavirus 2 **

## INTRODUCTION

**Fig. 1:**
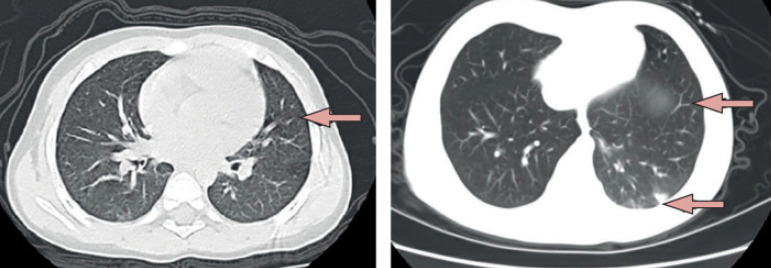
Chest computed tomography (CT) scans from two paediatric patients with coronavirus disease 2019[5]. Left: CT of 10-year-old boy showing multiple opacities in lower lobes of both lungs (arrow). Right: CT of 1.5-year-old girl showing multiple ground-glass opacities with a big patchy opacity in the right lung (arrows).

As of June 11, 2020, the severe acute respiratory syndrome coronavirus 2 (SARS-CoV-2) has been responsible for more than 3,296,245 infections and 414,974 deaths worldwide, but data regarding the epidemiologic characteristics and clinical features of infected children are limited^[1]^. The first confirmed pediatric case of SARS-CoV-2 infection was reported in Shenzhen, China, on January 20^[2]^. A study on 72,314 cases by the Chinese Center for Disease Control and Prevention showed that around 1% of the cases were children under ten years old^[3]^. Lu et al.^[4]^ analyzed the demographic data and clinical features of 171 cases under the age of 16 in Wuhan Children's Hospital. The results showed that the median age of the infected children was 6.7 years (1 day-15 years). The most common clinical manifestations were cough (48.5%), pharyngeal erythema (46.2%), and fever (41.5%). In contrast with infected adults, most infected children appear to have a milder disease progress. Asymptomatic infections were not uncommon^[5]^ (Figure 1).

Wu et al.^[3]^ reported that the current mortality of SARS-CoV-2 is 2.3%, but the mortality of patients with cardiovascular diseases and hypertension is 10.5% and 6.0%, respectively. At present, studies have shown that some infectious children have cardiovascular system injuries^[7]^. Wang et al.^[8]^ reported 31 cases of SARS-CoV-2 in children, of which four cases (15%) showed elevated serum creatine kinase (CK), with the highest peak reaching 360 U/L. Creatine kinase isoenzyme (CK-MB) also showed an increasing trend, reaching 85.3 U/L at the highest peak. This article summarizes the research progress of SARS-CoV-2 in China and discusses the mechanism and potential effects of cardiovascular injury in children.

## OVERVIEW OF SARS-CoV-2

Coronavirus is an enveloped, segmented positive-strand ribonucleic acid (RNA) virus, belonging to Coronaviridae and Nested Viruses. It is the largest positive-strand RNA virus known at present and is named for its virus shape, which is similar to crown shape under electron microscope. It can spread between humans and several other mammals^[9]^. Coronaviridae is divided into four genera of α, β, γ, and δ. There are currently seven known coronaviruses that can cause human infection, including human coronavirus 229E (or HCoV-229E), human coronavirus OC43 (or HCoV-OC43), severe acute respiratory syndrome coronavirus (SARS-CoV), human coronavirus NL63 (or HCoV-NL63), human coronavirus HKU1 (or HCoV-HKU1), Middle East respiratory syndrome coronavirus (MERS-CoV), and SARS-CoV-2^[10]^.

SARS-CoV-2 belongs to β genus of coronavirus, with round or oval shape and a diameter of 60~140 nm. Its gene characteristics are obviously different from SARS-CoV and MERS-CoV^[10]^, but its sequence homology with bat severe acute respiratory syndrome-like coronavirus ZC45 and ZXC (or bat-SL-CoVZC45 and bat-SL-CoVZXC) strains is more than 85%, suggesting that bats may be the original host of the virus^[12]^. A recent study revealed that SARS-CoV-2 has evolved into two subtypes, L and S, and their transmission ability and pathogenicity are quite different^[13]^.

## POSSIBLE MECHANISM OF SARS-CoV-2 INJURY TO CARDIOVASCULAR SYSTEM IN CHILDREN

On February 13, 2020, a clinical report issued by the American College of Cardiology, or ACC, pointed out that SARS-CoV-2 had certain effects on the heart. Patients with cardiovascular diseases have increased risks of complications and death^[14]^. At present, the evidence of myocardial injury in adult patients has been relatively clear. Huang et al.^[15]^ published 41 confirmed patients; five cases (12%) had myocardial injury, mainly manifested by an increase in high sensitivity cardiac troponin I, or hs-cTnI, level (> 28 pg/mL). Among 138 patients reported by Wang et al.^[8]^, 10 (7.2%) had acute myocardial injury and 23 (16.7%) had new arrhythmia; 36 severe patients were admitted to the intensive care unit (ICU), and the myocardial injury markers of patients admitted to ICU were significantly higher than those of non-ICU patients. Yang et al.^[16]^ reported that among 52 patients with severe SARS-CoV-2, 12 (23%) had myocardial injury, of which nine (17%) died. The latest version of the diagnosis and treatment plan for novel coronavirus in China clearly pointed out that some infected patients have increased myocardial injury markers such as CK and troponin, which requires vigilance against myocardial injury^[11]^.

Evidence of myocardial injury also exists in children with SARS-CoV-2 infection. The China's Expert Consensus on Diagnosis, Treatment and Prevention of Children's Novel Coronavirus Infection (2^nd^ Edition)^[17]^ mentioned that progressive increase of myocardial enzyme and lactate dehydrogenase often indicates aggravation or deterioration of the disease, and troponin is increased in some children. The ten cases reported by Cai et al.^[18]^ showed CK elevation during treatment. Zhang et al.^[19]^ described a pair of twin girls infected with SARS-CoV-2, the smaller of which showed an increase in lactate dehydrogenase and CK-MB. The first child with severe infection in China also suffered from cardiac injury during the progression of the disease, which was manifested by abnormal increase of CK-MB and N-terminal pro-brain natriuretic peptide^[20]^. The abovementioned literature shows that SARS-CoV-2 infection can cause certain damage to the heart both in adults and children. Although the mechanism by which SARS-CoV-2 affects the children's cardiovascular system is not completely clear at present, we speculate that SARS-COV-2 may play a role through the following aspects.

### Inflammation

The Novel Coronavirus Diagnosis and Treatment Plan (Trial 7^th^ Edition) points out that severe patients are often accompanied by elevated inflammatory factors^[11]^. The Expert Consensus on Diagnosis, Treatment and Prevention of novel coronavirus Infection in Children (2^nd^ Edition) also mentions that severe and critical children may be accompanied by elevated levels of inflammatory factors such as interleukin (IL)-6, IL-4, IL-10, and tumor necrosis factor alpha, or TNF-α^[16]^. The pathological anatomy results of the first adult SARS-CoV-2 death patient reported by Xu et al.^[21]^ show that there are a large number of inflammatory cells in the tissues of the patient's whole body, and a small amount of interstitial monocyte inflammatory infiltration can also be seen in the myocardium. It is reported that the serum levels of many inflammatory factors and C-reactive protein in SARS-CoV-2 adult patients are increased, and the expression level of inflammatory factors is closely related to the severity of the disease^[5,8,15]^. Studies have shown that inflammatory cell infiltration can aggravate myocardial cell apoptosis and even lead to malignant arrhythmia^[10]^.

### Angiotensin Converting Enzyme-2 (ACE-2)

**Fig. 2:**
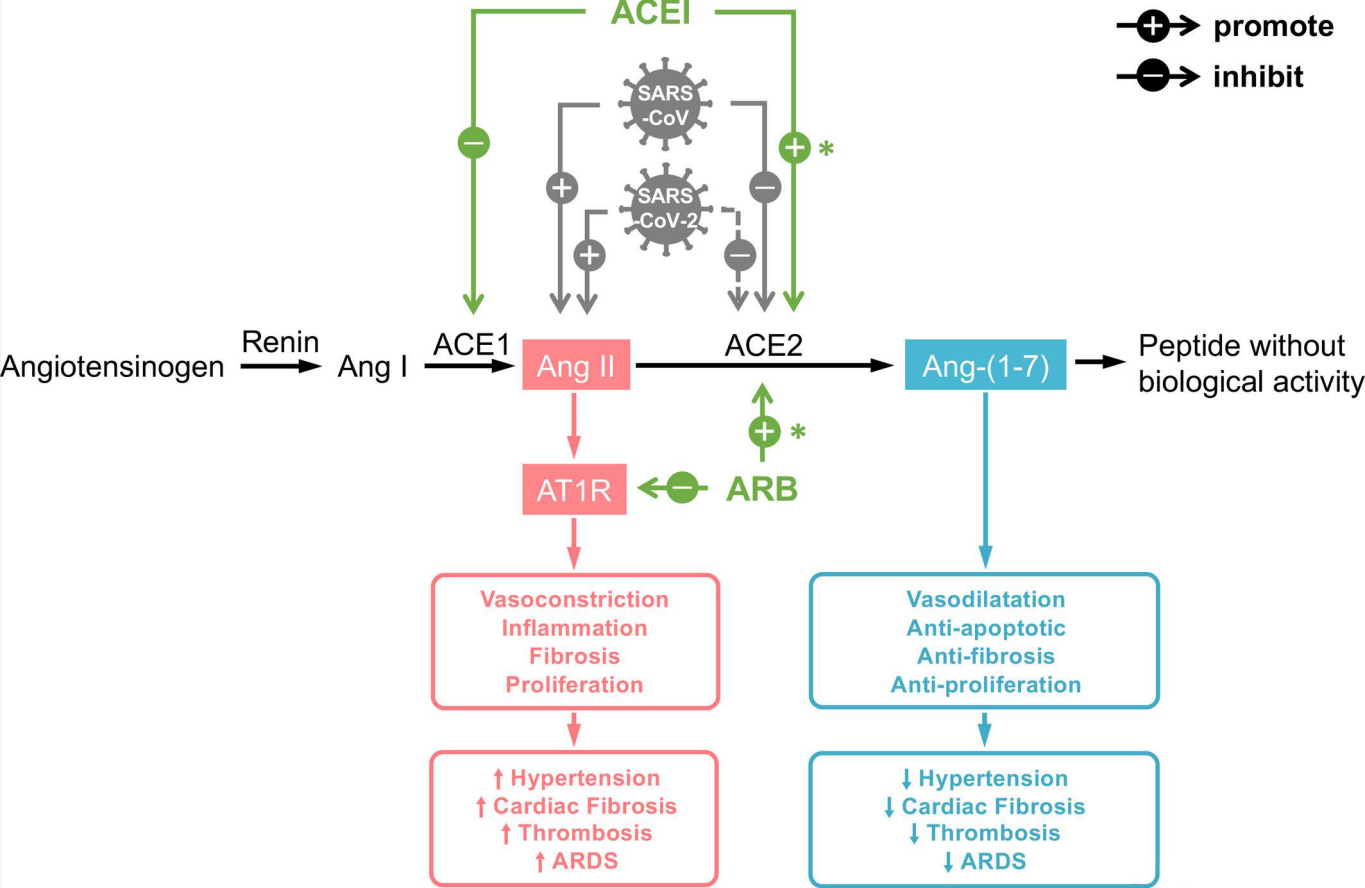
Severe acute respiratory syndrome coronavirus 2 (SARS-CoV-2) affects renin-angiotensin system and causes cardiovascular system changes[25]. ACE-1=angiotensin converting enzyme-1; ACE-2=angiotensin converting enzyme-2; ACEI=angiotensin converting enzyme inhibitor; Ang=angiotensin; ARB=angiotensin II receptor blocker; ARDS=acute respiratory distress syndrome; AT1R=angiotensin II type I receptor; SARS-CoV=severe acute respiratory syndrome coronavirus

Renin-angiotensin system is closely related to the progression of cardiovascular diseases such as atherosclerosis, myocardial fibrosis, and heart failure^[22]^. Angiotensin converting enzyme (ACE) can convert angiotensin (Ang) I into Ang II. ACE-2 is a homologue of ACE, which can hydrolyze Ang II into Ang I - Ang VII, thus achieving a series of cardiovascular protective effects such as vasodilation, inflammation inhibition, antioxidant stress, anti-fibrotic effects, antithrombotic effects, and anti-cardiovascular remodeling^[23]^. The expression of ACE-2 has high tissue specificity. It is mainly found on the surface of alveolar epithelial cells and is also highly expressed in heart, blood vessels, kidney, and gastrointestinal tissues^[24]^ (Figure 2).

Kuba et al.^[26]^ believe that SARS-CoV infection leads to a decrease in ACE-2 expression, which leads to an aggravation of the patient's condition. Oudit et al.^[27]^ found that SARS-CoV infection can lead to ACE-2-dependent myocardial injury, accompanied by a significant decrease in ACE-2 expression. In addition, an autopsy study of SARS-CoV patients found that 35% (7/20) of the patients had RNA of SARS-CoV virus in their myocardial tissues. At the same time, obvious macrophage infiltrating myocardial injury was found, and the presence of SARS-CoV in myocardium was also related to the downregulation of ACE-2 expression. Studies have shown that SARS-CoV-2 and SARS-CoV have similar effects and high affinity with ACE-2, suggesting that SARS-CoV-2 can damage myocardial cells via ACE-2 receptor^[28]^. Some scholars have analyzed the molecular structure of ACE-2, which exists as a dimer and has two conformational changes of opening and closing, and both conformations can combine with SARS-CoV-2, thus aggravating the damage of target cells^[29]^. However, more research is needed to confirm whether SARS-CoV-2 can directly attack myocardial cells.

### Hypoxemia

Severe SARS-CoV-2 infection can cause alveolar epithelium to form a transparent membrane, causing pulmonary ventilation and ventilation disorders, thus resulting in hypoxemia^[21]^. Hypoxia causes contraction of peripheral blood vessels, leading to an increase in cardiac preload and afterload. At the same time, hypoxia upregulates the expression of endocardial calcium channels in the left ventricle and changes ventricular repolarization time. This eventually led to a series of cell injuries, including apoptosis^[30]^. In addition, hypoxia can continuously stimulate the body to produce oxygen free radicals. Excessive oxygen free radicals increase endothelial growth factor and induce inflammatory reactions, such as inflammatory cell infiltration and cytokine release, which can also lead to vascular endothelial dysfunction and myocardial ischemia^[31]^.

### Stress Response

Children infected with SARS-CoV-2 (especially the older ones) have certain anxiety, and the fear and stress reactions of severe cases are more obvious. These emotional stress states will further damage children's immune function and induce cardiopulmonary injury^[32]^. Under stress, the body produces and releases catecholamines in large quantities and damages myocardium through direct cardiac toxicity and indirect microcirculation disturbance^[33]^. According to this mechanism, hypertension, myocardial injury, cardiac dysfunction, arrhythmia, and sudden cardiac death may occur as the condition of children with SARS-CoV-2 progresses.

## INFLUENCE OF SARS-CoV-2 ON CHILDREN'S CARDIOVASCULAR SYSTEM

### Acute Myocarditis

Virus infection is the most common cause of acute myocarditis in children. Studies have found that enteroviruses (especially the coxsackie virus), adenovirus, cytomegalovirus, Epstein-Barr virus, influenza virus, and parvovirus-B19 are all considered to be pathogenic viruses of myocarditis in children^[34]^. Previous literature has reported cases of acute myocarditis caused by MERS-CoV^[35]^. Rao et al.^[36]^ reported a nine-month-old infant with fulminant severe myocarditis induced by coronavirus infection. Liu et al.^[37]^ reported a 63-year-old male with fulminant myocarditis due to SARS-CoV-2 infection. According to the mechanism discussed above, SARS-CoV-2 infection may cause direct or indirect damage to the heart. At present, there is no case report of acute myocarditis caused by SARS-CoV-2 infection in children, but we still need to be vigilant. For children suspected of myocarditis, in addition to strengthening the detection of myocardial enzyme and myocarditis indexes and dynamic observation of electrocardiogram, cardiac magnetic resonance imaging is recommended for definite diagnosis under relatively stable condition. For children with acute myocardial injury, we should timely and reasonably apply drugs for myocardial protection.

### Hypertension

An epidemiological study shows that the incidence of primary hypertension in children is increasing with the change of human diet^[38]^. In 138 cases of adult SARS-CoV-2 infection reported by Wang et al.^[16]^, 58.3% of the severe patients were complicated with hypertension. Wu et al.^[3]^ found that the mortality rate of SARS-CoV-2 patients with hypertension was 6.0% after analyzing the data of 72,314 cases. At present, there is no research on children infected with SARS-CoV-2 and complicated with hypertension. However, referring to similar cases in adults, the risk of death for children with hypertension may increase.

Nowadays, there is still controversy over whether ACE inhibitor (ACEI) and angiotensin II receptor blocker (ARB) should be used in patients with hypertension. Ferraio et al.^[39]^ conducted a study; after they applied ACEI and ARB drugs to rats, the expression of ACE-2 in rat heart increased 4.7 times and 2.8 times, respectively. Therefore, the application of ACEI/ARB drugs has the risk of increasing SARS-CoV-2 infection. Some experts suggested that patients with SARS-CoV-2 complicated with hypertension should suspend ACEI/ARB therapy and switch to calcium antagonists, α-receptor antagonists, etc. to control blood pressure^[40]^. However, there are also views that the application of ACEI/ARB drugs can reduce the inflammatory reaction in the lungs of SARS-CoV-2 patients, thus reducing the mortality of patients^[41]^. Liu et al.^[35]^ reported a significant increase in Ang II level in patients' plasma, suggesting that ACEI/ARB can be used to treat SARS-CoV-2 patients. Peng et al.^[42]^ found that ACEI/ARB did not affect the morbidity and mortality of SARS-CoV-2 patients with cardiovascular diseases. Therefore, the blood pressure of children with SARS-CoV-2 complicated with hypertension should be closely monitored, and further research is needed on whether ACEI/ARB drugs should be applied.

### Heart Failure

Heart failure refers to low ventricular pumping function and/or filling function. Subsequently, pulmonary congestion occurred due to decreased myocardial contractility and excessive activation of neurohumoral regulation mechanisms. For children with pulmonary congestion, the incidence of pulmonary infection is higher^[43]^. Chen et al.^[44]^ reported 99 cases of adult SARS-CoV-2 patients, including one 61-year-old male who had no underlying diseases before, but died of severe heart failure during treatment. Peng et al.^[42]^ reported 112 cases of SARS-CoV-2 complicated with cardiovascular diseases, 40 of which were complicated with heart failure, and 13 died at last. This showed that the prognosis of patients with heart failure infected with SARS-CoV-2 is worse. At present, there is no relevant research on children, but referring to the clinical experience of adult SARS-CoV-2 cases, it is not excluded that children with heart failure have the possibility that heart failure rapidly aggravates after being infected with SARS-CoV-2, and then develops into severe or critical illness^[33]^. Because children with a history of heart failure are already in a state of water and sodium retention and immunosuppression, they are prone to lung infection. Once pulmonary infection happens, it can induce pulmonary hypertension and increase right ventricular load, thus aggravating cardiac function damage. Besides, the lung produces gas exchange disorder, which leads to myocardial cell injury caused by hypoxemia^[45]^. Children with SARS-CoV-2 often have fever symptoms, which can lead to sympathetic excitation, increase heart rate and myocardial oxygen consumption, reduce cardiac output, and further aggravate heart failure. In addition, older children with heart failure already have psychological pressure of long-term illness. When infected with SARS-CoV-2, additional psychological pressure will inevitably be caused to the children, and these psychological stress processes will promote the release of catecholamine in large quantities, further aggravating the damage to myocardium^[31]^. Therefore, it is particularly important to prevent children with congenital heart disease from viral infection. Once infected with SARS-CoV-2, the total volume of transfusion should be carefully controlled, and children should be closely monitored when transfusion is necessary.

## CONCLUSION

Up to now, the number of children infected with SARS-CoV-2 is relatively small. SARS-CoV-2 can cause cardiovascular system injury in children, and its pathogenesis is more complex. Children with cardiovascular diseases will face greater risks after contracting SARS-CoV-2. Therefore, the most important thing is to protect children from SARS-CoV-2 infection.

**Table:** 

Authors' roles & responsibilities	
SW	Substantial contributions to the acquisition of data for the work; drafting the work; final approval of the version to be published
YZ	Substantial contributions to the acquisition of data for the work; drafting the work; final approval of the version to be published
LW	Revising the work; final approval of the version to be published
